# Effect of probiotic bacteria on porcine rotavirus OSU infection of porcine intestinal epithelial IPEC-J2 cells

**DOI:** 10.1007/s00705-022-05510-x

**Published:** 2022-07-06

**Authors:** Danielle Leblanc, Yves Raymond, Marie-Josée Lemay, Claude P. Champagne, Julie Brassard

**Affiliations:** Saint-Hyacinthe Research and Development Centre, Agriculture and Agri-Food Canada, 3600 Casavant Boulevard West, Saint-Hyacinthe, QC J2S 8E3 Canada

## Abstract

Rotavirus infections in nursing or post-weaning piglets are known to cause diarrhea, which can lead to commercial losses. Probiotic supplementation is used as a prophylactic or therapeutic approach to dealing with microbial infections in humans and animals. To evaluate the effect of probiotic bacteria on porcine rotavirus infections, non-transformed porcine intestinal epithelial IPEC-J2 cells were used as an *in vitro* model, and three different procedures were tested. When cells were exposed to seven probiotics at concentrations of 10^5^, 10^6^, or 10^7^ CFU/mL for 16 h and removed before rotavirus challenge, infection reduction rates determined by flow cytometry were as follows: 15% (10^6^) and 18% (10^5^) for *Bifidobacterium longum* R0175, 15% (10^7^) and 16% (10^6^) for *B*. *animalis lactis* A026, and 15% (10^5^) for *Lactobacillus plantarum* 299V. When cells were exposed to three selected probiotic strains for 1 h at higher concentrations, that is, 10^8^ and 5 × 10^8^ CFU/mL, before infection with rotavirus, no significant reduction was observed. When the probiotic bacteria were incubated with the virus before cell infection, a significant 14% decrease in the infection rate was observed for *B. longum* R0175. The results obtained using a cell-probiotics-virus platform combined with flow cytometry analysis suggest that probiotic bacteria can have a protective effect on IPEC-J2 cells before infection and can also prevent rotavirus infection of the cells.

**Supplementary Information:**

The online version contains supplementary material available at 10.1007/s00705-022-05510-x.

## Introduction

In swine production, piglets experience significant stress at weaning, a period when their immune system is still immature, which makes them susceptible to infections. Rotavirus causes profuse watery diarrhea that can lead to dehydration and malabsorption, particularly in young animals, and cause important economic losses [1, 2]. Rotavirus is transmitted via the fecal-oral route, and fecal shedding of the virus facilitates its transmission. Rotavirus groups A, B, and C are the major groups associated with gastrointestinal disease in pigs [1, 3]. As in humans, rotavirus vaccines are available to prevent infections in pigs caused by major group A rotavirus (RVA) strains [1]. Because of the wide genetic diversity among rotaviruses and the emergence of new circulating strains, the efficacy of vaccines may decrease over time. In addition to their therapeutic uses, antibiotics have been used to promote growth or to provide protection against certain diseases and symptoms, including diarrhea [4]. However, few antimicrobial agents are available for use in controlling porcine viruses [5], and antibiotics are known to disrupt the ecology of the intestinal microbiome. In general, the use of antibiotics tends to be limited in order to avoid antibiotic resistance in pathogenic microorganisms.

There is substantial evidence for the benefits of using probiotic bacteria to promote the health of the digestive system and to prevent infections or mitigate the symptoms of intestinal illness [2, 4, 6, 7]. Along with vaccines, probiotics provide an additional tool for protecting animals from infections as well as a partial alternative to the use of antibiotics as a growth promoter [4].

In pregnant and lactating sows, piglets, and fattening sows, probiotics can be used to support the digestive system, decrease stress, and reduce the risk of infections, leading to better growth performance [4, 8]. Their mode of action in animals has not been fully elucidated but may include producing antimicrobial substances, excluding microbial pathogens by blocking adhesion sites, preventing microbial pathogens from attaching to the epithelium, improving the barrier against microbial invasion by tightening the junctions between intestinal cells, increasing mucus production, which prevents pathogen adhesion, and changing the composition of intestinal flora [7, 9–11]. Hosts can benefit from the immunomodulatory properties (activation of macrophages, increased secretion of immunoglobulins) associated with the presence of probiotics [10, 12]. As feed additives, probiotics can promote the general health of piglets by helping them develop and maintain a healthy gut microflora. They can also help re-establish the intestinal flora of animals following antibiotic treatment [13].

The genus *Lactobacillus* has been divided into many groups [14], which will be referred to collectively as lactobacilli. *Bifidobacterium* and lactobacilli are normally found in the intestinal microflora and are among the most commonly used probiotic bacteria in animal nutrition [4, 13, 15]. Several *in vitro* studies have demonstrated the effectiveness of probiotics: *Bifidobacterium* (B.) *breve* MCC1274 and *B. infantis* MCC12 were found to decrease bovine and porcine rotavirus infection of porcine intestinal epithelial cells [16], *Bifidobacterium longum* subsp. *infantis* was found to inhibit human rotavirus strain Wa replication and infection of MA104 and HT-29 cells [17], *Bifidobacterium adolescentis* DSM 20083 and *Lacticaseibacillus* (*Lcb.*) *casei* Lafti L26-DSL were found to interfere with infection by a rhesus monkey rotavirus strain [18]. *Lacticaseibacillus rhamnosus* strain CNCM I-3690 was found to protect the gut barrier of mice by modulating the production of mucus and the mechanisms that protect cells [19]. In a study by Park et al. [20], a total of 57 infants infected with rotavirus were given probiotic formula containing *B. longum* BORI and *Lactobacillus (Lb.*) *acidophilus* AD031 or a placebo. Although the differences between the groups were not statistically different, the symptoms (duration of fever and frequency of diarrhea and vomiting) were reduced by the probiotic treatment [20]. Selle and Klaenhammer reported that *Lb*. *gasseri* helped maintain gut homeostasis in humans in addition to having various other health benefits [21].

Using gnotobiotic pigs, Kandasamy et al. [22] compared the effects of administering *Lcb. rhamnosus* strain GG and *B. animalis lactis* Bb12 strains in combination with an attenuated human rotavirus strain WA vaccine to piglets, with or without probiotics, and then challenging them with human rotavirus (HRV). In probiotic-colonized piglets, there was an increase in intestinal IgA titers, which was correlated with less-intense symptoms of diarrhea and a favourable modulated B-cell response to the vaccine. Mao et al. [23] compared various parameters in 11-day-old weaned piglets after ingestion of rotavirus OSU. In that study, the animals with dietary supplementation of *Lcb. rhamnosus* GG showed reduced severity of induced diarrhea, which appeared to be linked to increased mucosal barrier efficiency combined with reduced virus multiplication and stimulation of the immune response.

Before porcine intestinal cells came into use, *in vitro* models used cells from other animal species that were less representative of the reaction that takes place in the intestine of piglets. IPEC-J2 cells were subsequently characterized and suggested as an *in vitro* model for studying microbiological interactions with the porcine epithelium [24–30]. IPEC-J2 cells are non-transformed intestinal epithelial cells derived from the jejunum of a neonatal pig. Closely related to human intestinal cells and of noncancerous origins, they are used for *in vitro* experimentation with probiotics [31, 32]. Liu et al. [28] first reported the use of this *in vitro* model to study the interactions of *Lactobacillus* (*Lb.*) *acidophilus* and *Lcb. rhamnosus* GG with rotavirus infection and the innate immune response of IPEC-J2 cells.

Earlier studies demonstrated that *Lcb. rhamnosus* strains CRL 1505 and CRL 1506 can modulate innate immunity and increase cytokine production after simulation of viral infection with poly(I:C) of non-transformed epithelial porcine cells (IEC) [33]. Furthermore, *Lactiplantibacillus* (*Lpb*) *plantarum* strain CGMCC1258 isolated from healthy infants was found to protect IPEC-J2 cells against *E. coli* ETEC strain K-88 [34].

It has been reported that the beneficial effect of probiotics on animal health may be strain-specific [9, 15, 28, 33]. Hence, there is a need to identify more strains that have antiviral attributes and the conditions that promote their effectiveness.

Using an *in vitro* model, this study aimed to provide valuable insights into the specific antiviral properties of seven probiotic strains of lactobacilli and *Bifidobacterium* that protect against porcine rotavirus infection of IPEC-J2 cells.

## Materials and methods

### Cell culture conditions

Intestinal epithelial IPEC-J2 cells (DSMZ German collection, Braunschweig, Germany) were grown in Dulbecco’s modified Eagle medium (GM: DMEM/Ham F12 [50:50]; Wisent Bioproducts, St-Bruno, QC, Canada) containing L-glutamine and 15 mM HEPES and supplemented with 5% inactivated fetal bovine serum (Wisent Bioproducts), 1% penicillin-streptomycin (Wisent Bioproducts), 5 ng of epidermal growth factor (EGF) (Wisent Bioproducts) per mL, and 1% insulin-transferrin-selenium premix (ITS) (Corning, NY, USA).

### Production of rotavirus OSU

IPEC-J2 cells were grown in the medium described above in F175 flasks for 5 days at 37 °C and incubated for 1 h with agitation with trypsin-treated (30 min) rotavirus OSU (VR-892, ATCC, Manassas, VA, USA) diluted in maintenance medium (MM: DMEM/F12, L-glutamine, EGF, ITS) and supplemented with cholesterol (1:500) (Sigma-Aldrich, Oakville, ON, Canada; MMC: maintenance medium with cholesterol) for cell culture. Infection took place at a multiplicity of infection (MOI) of 0.75. Viruses were removed, and the cell overlay was washed with maintenance medium. MMC was added, followed by a 5- to 6-day incubation at 37 °C. After three freeze-thaw cycles, viruses were recovered in the supernatant following centrifugation at 4,000 × *g* for 20 min (Sigma 4K15; QIAGEN, Montreal, QC, Canada), filtered through a 0.22-µm membrane, and then kept at -80 °C. The viral suspension was concentrated using 100 kDa Amicon Ultra-15 centrifugal filter units (EMD Millipore).

### Preparation of bacterial strains

Experiments were carried out using *Lcb. rhamnosus* R0011, *B. longum* R0175, and *Lpb. plantarum* 299V (Lallemand Health Solutions, Montreal, QC, Canada) as well as *Lcb. paracasei* A234, *B. lactis* A026, and *Lb. gasseri* A237 (Biena, Saint-Hyacinthe, QC, Canada) and *Lcb. rhamnosus* GG (isolated from a commercial source). Stock cultures of probiotics were obtained by mixing MRS-grown (Difco, Detroit, MI, USA) bacterial suspensions with sterile MRS containing 15% (w/v) glycerol (Sigma, St. Louis, MO, USA) in a 1:5 ratio. The cell suspensions were then distributed in 1-mL cryovials (Nalgene, Rochester, NY, USA) and frozen at −80 °C. Fresh liquid inocula of probiotic bacteria were prepared by adding 1 mL of thawed stock culture to 100 mL of MRS-AC medium and incubated at 37 °C until a pH of 4.5 was reached. The MRS-AC medium was prepared by adding 1 mL of a filter-sterilized solution of 10% (w/v) ascorbic acid (Sigma-Aldrich) and 5% (w/v) l-cysteine (Sigma-Aldrich) to 100 mL of sterile MRS. The fresh cultures were centrifuged for 15 min at 10,000 × *g* (Beckman Model J-20 XPI, Palo Alto, CA, USA). Pellets were washed twice with Dulbecco's phosphate-buffered saline (D-PBS) (Wisent, Boucherville, QC, Canada), and bacterial cultures were diluted in buffered maintenance medium (BMM: DMEM/F12 with 15 mM HEPES and 1.3 g of NaH_2_PO_4_ and 1.7 g of Na_2_HPO_4_ per liter) to obtain an initial concentration of 10^8^ colony-forming units (CFU)/mL before further dilution to 10^5^, 10^6^, or 10^7^ CFU/mL, or to obtain an initial concentration of 10^9^ CFU/ml for further dilution to 10^8^ or 0.5 × 10^8^ CFU/mL.

### Titration of rotavirus OSU

In 96-well plates, 200 µL of IPEC-J2 cells in growth medium at a concentration of 1.0 × 10^5^ cells/mL were incubated for 3 days at 37 °C in 5% CO_2_. The medium was then removed and replaced by medium without serum, and the cells were incubated again for 24 h. The cells were washed twice with 200 µL of MM and 100 µL of MM containing 1 µg of trypsin per mL. After activation by trypsin as described previously, virus suspensions were serially diluted in MM. A volume of 100 µL of viral inoculum was added to the designated wells. Plates were incubated at 37 °C in 5% CO_2_ for 1 h. After two washes, 200 µL of GM was added and the plates were further incubated for 18 to 24 h. The plates were then washed with PBS containing 5% normal donkey serum (NDS), and the cells were fixed with 200 µL of 80% acetone for 30 min at 4 °C. After two washes with 200 µL of PBS, cells were incubated for 1 h at ambient temperature with PBSTD (PBS + 0.05% Tween 20, 5% NDS). A total of 50 µL of sheep anti-porcine rotavirus OSU serotype V polyclonal IgG was added at a pre-determined concentration and incubated for 1 h at 37 °C. After two washes in PBSTD, 50 µL of Alexa Fluor 647–conjugated donkey anti-sheep antibodies were added to each well, and the plates were further incubated for 45 min at 37 °C. Cells were washed four times with PBSTD before visualization using fluorescence microscopy (EVOS IF combined with Cy5 cube). The 50% endpoint titre (TCID_50_) was calculated using the formula proposed by Ramakrishnan [35].

### Probiotic pre-treatment of an IPEC-J2 monolayer followed by viral infection

**Fig. 1:**
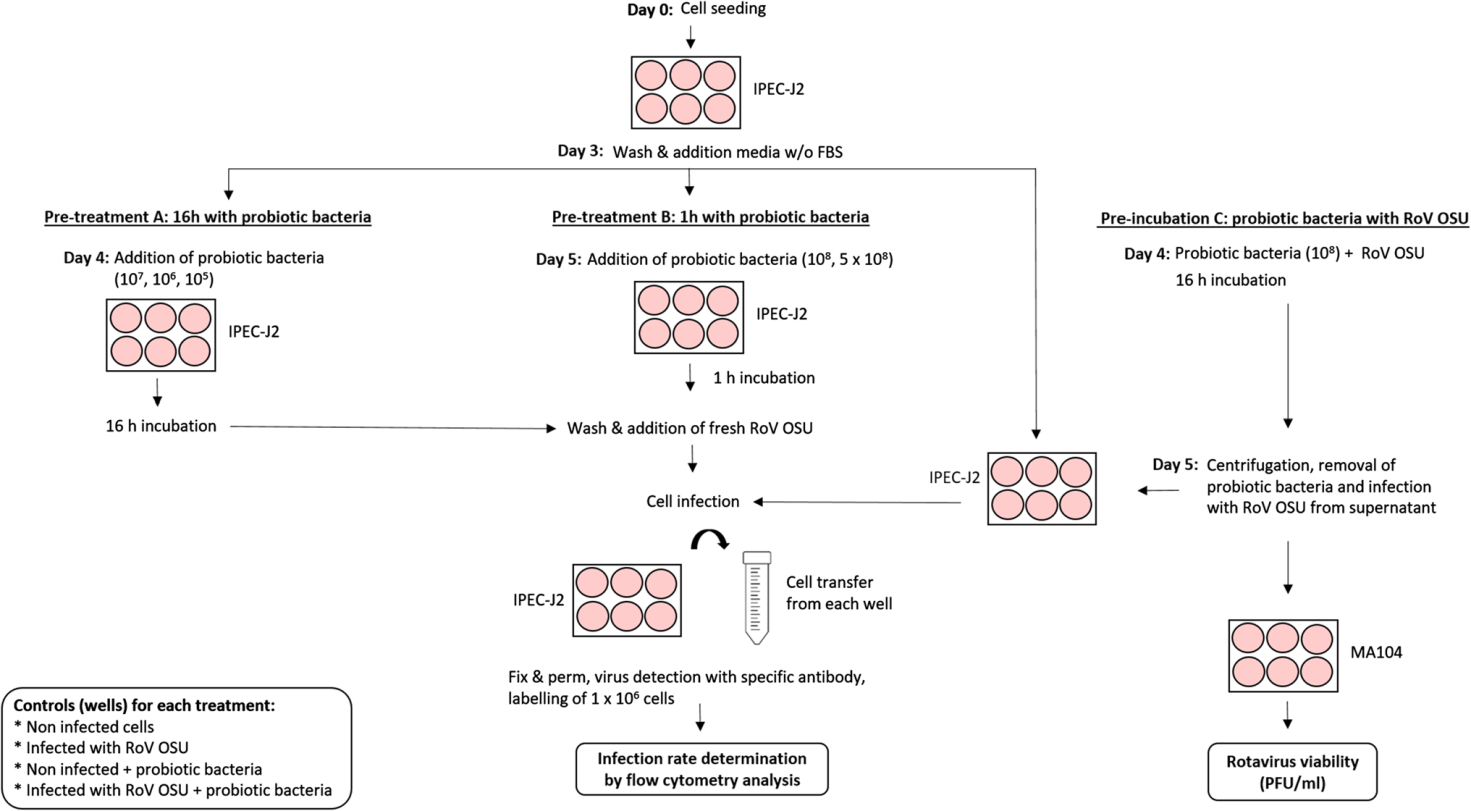
Schematic representation of the experimental design

On day zero, 6-well plates were seeded at a density of 3 × 10^5^ IPEC-J2 cells/well and incubated for 3 days at 37 °C in 5% CO_2_. In one series of assays (Fig. 1; Pre-treatment A), cells were washed with PBS and incubated for 24 h in MM. On day four, 3 mL of fresh diluted bacterial suspensions (10^5^, 10^6^, or 10^7^ CFU/mL) of *Lcb. rhamnosus* R0011, *B. longum* R0175, *Lpb. plantarum* 299V, *Lcb. paracasei* A234, *B. lactis* A026, *Lb. gasseri* A237, and *Lcb. rhamnosus* GG were added to each well, and the plates were further incubated for 16 h (Fig. 1; Pre-treatment A). After probiotic pre-treatment on day 5, the medium was discarded and the cells were washed twice before infection. Rotavirus activation was performed by adding a solution of trypsin type IX-S (Sigma-Aldrich) at 2 mg/mL in PBS 7.4 to porcine rotavirus OSU (ATCC, VR-892) (1:200), followed by incubation at 37 °C for 30 min. The cells in each well of a 6-well plate were infected with 1 mL of activated rotavirus (MOI: 26) diluted in BMM supplemented with cholesterol (1:500) (BMMC) and incubated at 37 °C in 5% CO_2_ with constant agitation for 1 h for virus attachment. Cells were washed twice with 2 mL of warmed PBS, followed by 2 mL of MM. The plates were incubated again for 4 h at 37 °C in 5% CO_2_ after the addition of 3 mL of BMMC containing 0.5 µg of trypsin IX-S per mL.

In a second series of assays (Fig. 1; Pre-treatment B), IPEC-J2 cells were exposed for 1 h to the selected strains, *B. longum* R0175, *B. lactis* A026, and *Lpb. plantarum* 299V, at 1 × 10^8^ and 5 × 10^8^ CFU/mL, on day 5 before virus infection, as described for Pre-treatment A. For both series of pre-treatments (A and B), controls (probiotic -/virus-; probiotic +/virus-: probiotic-/virus+) were included and treated in the same manner. One experiment was carried out by mixing the three strains. In this case, the inoculum was composed of each strain at 1 × 10^8^ CFU/mL.

### Pre-incubation of rotavirus OSU with probiotic bacteria followed by infection of an IPEC-J2 monolayer

These assays correspond to “Pre-incubation C” in Fig. 1. A total of 10^8^ CFU of each strain of probiotic bacteria was mixed with 1.5 × 10^8^ PFU of rotavirus OSU and agitated at 37 °C for 16 h (Fig. 1; pre-incubation C). Samples were centrifuged (16,000 × *g* for 5 min) and filtered through a 0.22-µm membrane. They were then activated by trypsin treatment and used to infect IPEC-J2 cells as described before (Fig. 1; pre-treatments A and B). Controls without virus and probiotics were included and treated in the same manner.

### Rotavirus infectivity of MA104 cells

The impact of probiotic pre-treatment on rotavirus viability (Fig. 1; pre-incubation C) was evaluated using MA104 cells. In a final volume of 1 mL, 1.5 × 10^8^ PFU of rotavirus OSU was mixed with probiotic bacteria at 10^8^ CFU/mL in BMM and incubated at 37 °C in 5% CO_2_ for 16 h. After centrifugation (16,000 × *g* for 5 min) and filtration through a 0.22-µm membrane, virus viability was determined by plaque assay according to the method of Arnold et al. [36] and compared with a virus suspension without probiotic bacteria. Controls without virus and probiotics were included and treated in the same manner.

### Determination of rate of infection of IPEC-J2 cells by rotavirus OSU, using flow cytometry analysis

After infection, IPEC-J2 cell monolayers were washed with 500 µL of PBS and added to the removed supernatants to recover all cells from each well. Cells were detached by treatment with 1 mL of 0.05% trypsin-0.53 mm EDTA. The wells were washed with 500 µL of PBS containing 5% normal donkey serum (NDS) (Wisent Bioproducts), and the liquids were pooled to include all of the cells. The cells were then centrifuged at 350 × *g* for 5 min (Sigma 4K15, QIAGEN), and after viability evaluation, 1 × 10^6^ cells were resuspended in 100 µL of PBS containing 5% NDS and incubated for 30 min before the fixation step, which was performed according to the manufacturer’s protocol (Fix & Perm Cell Permeabilization Kit, Thermo Fisher, Burlington, ON, Canada). Briefly, 100 µL of fixation medium was mixed with the cells, which were incubated at room temperature for 15 min and then washed with 3 mL of PBS containing 5% NDS (350 ×* g* for 5 min). Afterward, they were resuspended in 100 µL of permeabilization medium and mixed with 50 µL of sheep anti-porcine rotavirus OSU serotype V polyclonal IgG (American Research Products Inc., Waltham, MA, USA) at a pre-determined concentration. The mixtures were incubated at 4 ºC for 16 h in the dark, and the cells were washed with 3 mL of PBS containing 5% NDS, centrifuged (350 × *g* for 5 min), and mixed with 50 µL of Alexa Fluor 647–conjugated donkey anti-sheep antibody (Thermo Fisher) or Alexa Fluor 647-AffiniPure F(ab)2 donkey anti-sheep IgG antibody (Jackson ImmunoResearch Laboratory, West Grove, PA, USA) suspended in PBS containing 5% NDS. Mixtures were incubated at 4 °C for 45 min in the dark. Cells were washed with PBS before propidium iodide (PI) was added at 1 mg/mL (2 µL in 300 µL of cell suspension). Sample fluorescence was evaluated using a Beckman CytoFLEX S flow cytometer at 30 µL/min for 6 min with an excitation/emission filter combination of AF647: 638 nm/660 nm (20 nm) and PI: 561 nm/610 (20 nm). Acquisition thresholds were set at 10,000 scale on SSC-H and 10,000 scale on FSC-H. Data analysis was performed using Beckman Kaluza software. Compensation calculated with fluorescence minus one (FMO) controls was applied to all results analyzed.

### Statistical analysis

Experiments were carried out in triplicate. Results are expressed as the mean of infection rate reduction (%) ± standard error of the mean (SEM). Student’s *t*-test was used to compare mean infection rates for samples treated with probiotics and samples not treated with probiotics. Values were considered significant at *P* <0.05. Statistical analysis was performed using Microsoft Excel with the Analysis ToolPak.

## Results

**Fig. 2:**
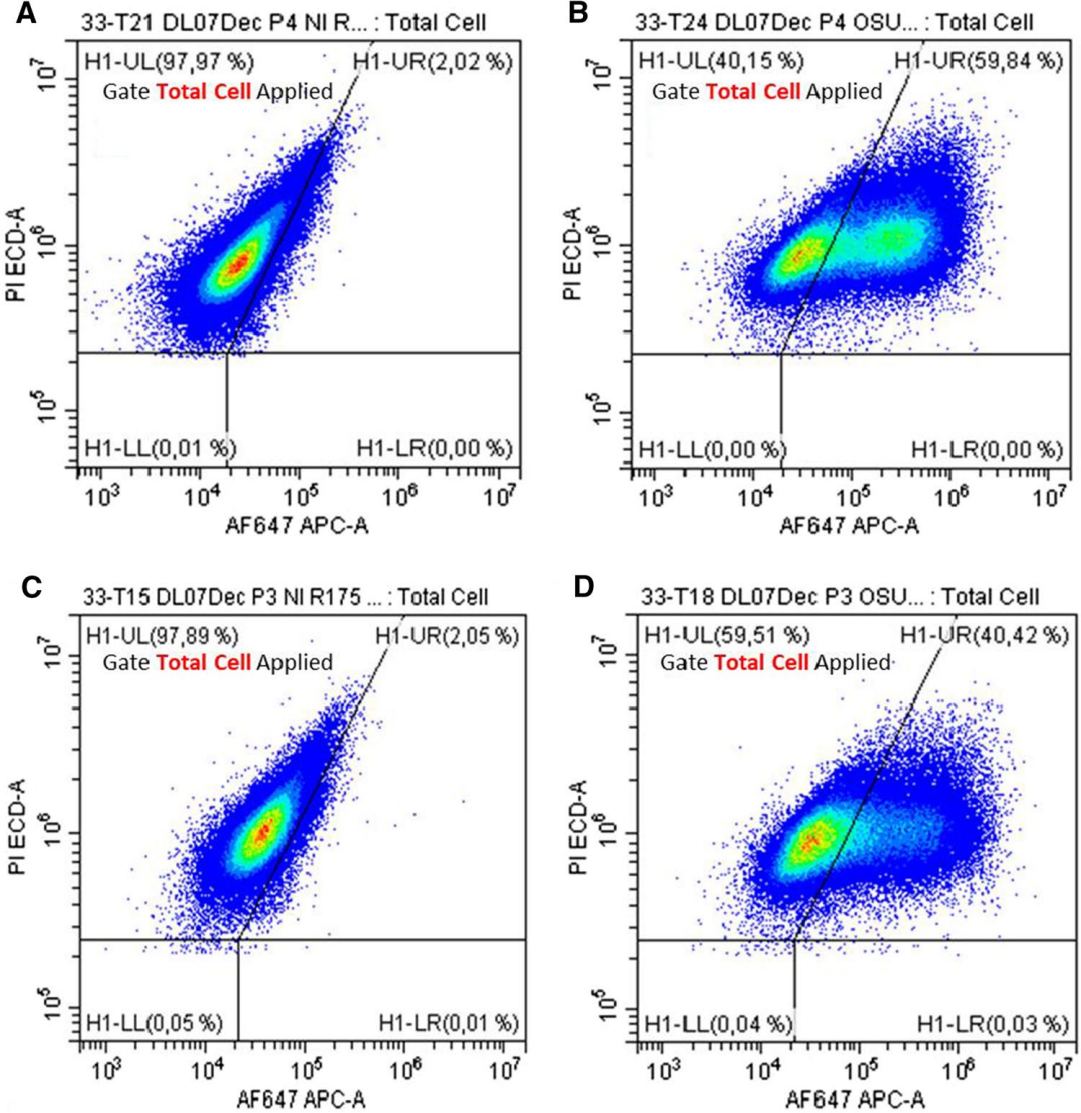
Graphic representation of Alexa Fluor–647 labelled IPEC-J2 cells analyzed using cytometry analysis after infection with rotavirus OSU for 4 h. (A) Uninfected. (B) Infected. (C) Uninfected with *B. longum* R0175 pre-treatment. (D) Infected with *B. longum* R0175 pre-treatment. Rotavirus-infected cells were identified as those exceeding the AF-647 fluorescence of uninfected cells treated simultaneously under the same experimental conditions. Results were expressed as the percentage of AF-647-positive cells in a sample

The use of the IPEC-J2 *in vitro* model to evaluate the effectiveness of probiotic bacteria at inhibiting porcine rotavirus infection provides a controlled environment for rapidly identifying and selecting strains with a high potential for infection prevention. In this study, the conditions for virus infection, cell preparation, cell labelling, and cytometry analysis were established for the *in vitro* model in order to examine the effect of the presence of probiotic bacteria on viral infection. Since rotavirus strain OSU is lytic, the goal was to obtain a maximum rate of infection while avoiding cell lysis. Conditions were set at 1 hour for the infection period, followed by a 4-hour incubation time, combined with a high-infectious-dose inoculum. For pre-treatments A and B (Fig. 1), the conditions selected to expose the IPEC-J2 cells to probiotics (medium, inoculation level, and incubation time) did not result in post-incubation pH values lower than 6.0, nor did they reduce the viability of the IPEC-J2 cells by more than 2% (Fig. 2 for *B. longum* R0175 – data not shown for all other strains). It should be kept in mind, when examining data from the literature, that some conditions result in enough acidification of the medium to create cytotoxicity [28].

Subsequently, a protocol for detachment, labelling of infected cells (fixation and permeabilization, blocking agents, nucleic acid dye), and quantification by flow cytometry was developed. For analysis, on a count versus PI-A graph (Supplementary Fig. S1A and D), a first region of interest P4 (presence of cells labeled marked with PI) was determined and was applied on a SSC-A versus FSC-A graph (Supplementary Fig. S1B and E). From that graph, the region of interest (cell total) was delimited and applied on a PI-A versus AF647-A graph. The regions of interest were then created (H1-UL: uninfected cells, H1-UR: infected cells) (Supplementary Fig. S1C and F).

Rotavirus-infected cells were identified as those exceeding the Alexa Fluor 647 (AF-647) fluorescence of uninfected cells treated simultaneously under the same experimental conditions. Results were expressed as a percentage of AF-647-positive cells in a sample (Supplementary Fig. S1C and F). For each condition of infection to be evaluated, a control treated in the same manner but without the virus was used to delineate the zone of Alexa Fluor 647 fluorescence of uninfected cells on the flow cytometry dot plot of the infected samples. Each sample was also labelled using nucleic-acid-specific propidium iodide dye to identify cells (infected and uninfected). Probiotic bacteria were used at concentrations and under conditions limiting acidification of the medium, to avoid a negative impact on the monolayers.

### Effect of probiotic pre-treatment on infection of IPEC-J2 cells by rotavirus

IPEC-J2 cells were exposed for 16 h to concentrations of probiotic bacteria ranging from 10^5^ to 10^7^ CFU/mL (Fig. 1; pre-treatment A). Before the fixation and permeabilization step, and to ensure that the presence of bacteria did not affect the cells, the average IPEC-J2 cell viability was estimated, with trypan blue staining between 95% and 100% of the cells. Also as can be seen in Fig. 2 (A and C), the probiotic bacteria did not reduce the viability of IPEC-J2 cells. This rules out the possibility that variations in virus infection could be due to reduced viability of the animal cells. To demonstrate the effect of probiotic bacteria on IPEC-J2 cells infected with rotavirus OSU, the strain *B. longum* R0175 was inoculated at 10^5^ CFU/mL (Fig. 2).

**Fig. 3:**
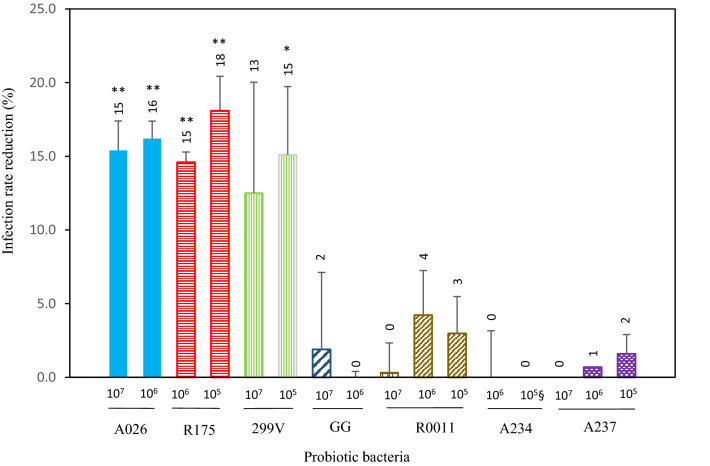
Infection rate reduction expressed as the difference between IPEC-J2 cells infected with rotavirus OSU (%) minus IPEC-J2 cells pre-treated for 16 h with probiotic bacteria before infection with OSU (%) (Pre-treatment A). Values represent the mean (± SEM) of three separate experiments. * *p* <0.05; ** *p* <0.01; §, mean of two assays in one experiment

Bacterial concentrations were selected for the assays in order to determine whether a correlation existed between the concentration of probiotic bacteria and the reduction in infection rate. The results of the cell infection assays (Fig. 3) showed a significant decrease in the infection rate of 15% (*p* = 0.001) and 18% (*p* = 0.008) for *B. longum* R0175 at concentrations of 10^6^ and 10^5^, respectively. At a concentration of 10^7^ CFU/mL, the buffering conditions did not allow the pH level to be maintained above 6.0; hence, the results were excluded (data not shown). *Bifidobacterium animalis lactis* A026 inoculated at 10^7^ and 10^6^ CFU/mL reduced rotavirus infection rates by 15% (*p* = 0.009) and 16% (*p* = 0.003), respectively. For both strains, no significant difference was found between the results for the two concentrations of the same probiotic bacteria (*B. longum* R0175, *p* = 0.175); *B. animalis lactis* A026, *p* = 0.332). At 10^5^ CFU/mL, *Lpb. plantarum* 299V also decreased the infection rate by 15% (*p* = 0.041) (Fig. 3), but no significant difference (*p* = 0.256) was found between the two bacterial concentrations tested. This can be attributed to the greater variability in results obtained with strain 299V under our experimental conditions (Fig. 3). A small reduction or no reduction in the rotavirus OSU infection rate for the concentrations assayed was observed with *Lcb. rhamnosus* GG, *Lcb. rhamnosus* R0011, *Lcb. paracasei* A234, and *Lb. gasseri* A237 (Fig. 3).

IPEC-J2 cells were exposed for 1 h to 1 × 10^8^ and 5 × 10^8^ CFU/mL (Fig. 1; pre-treatment B) of strains *B. animalis lactis* A026, *B. longum* R0175, and *Lpb. plantarum* 299V, which showed significant reductions in the infection rate in the 16-h pre-treatment at concentrations varying from 10^5^ to 10^7^ CFU/mL. Two higher concentrations (1 × 10^8^ and 5 × 10^8^ CFU/mL) were also applied before incubation with IPEC-J2 cells (Supplementary Fig. S2). The purpose of this step was to determine whether an increased bacterial concentration in contact with IPEC-J2 cells could produce a rapid reduction in rotavirus infection. The short (1 h) incubation period was selected to avoid acidification of the medium and loss of IPEC-J2 cell viability. A mixture of the three strains at 1 × 10^8^ CFU/mL was also tested (Supplementary Fig. S2). No reduction in the infection rate was observed under those conditions.

### Effect of probiotic bacteria and rotavirus pre-incubation on viral infectivity

**Fig. 4:**
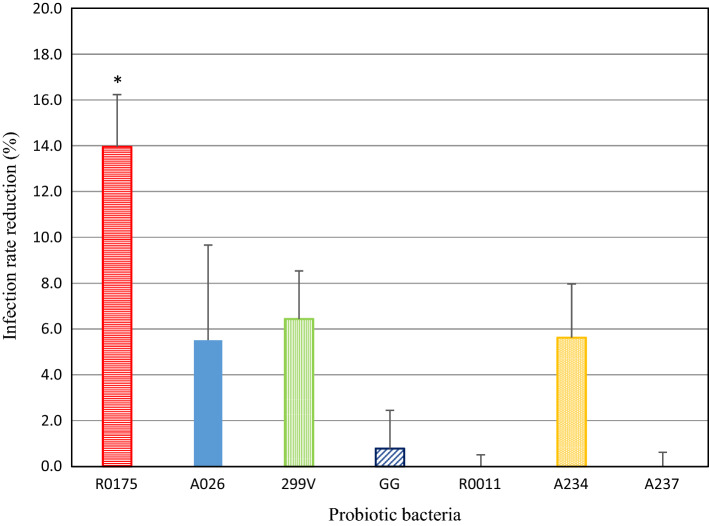
Infection rate reduction expressed as the difference between IPEC-J2-infected cells with OSU (%) minus IPEC-J2-infected cells (%) with OSU previously incubated 16 h with probiotic bacteria (1 × 10^8^ CFU/mL). Bacteria were removed by centrifugation and filtration prior to infection (Pre-Incubation C). Values represent the mean (± SEM) of three separate experiments. *p < 0.05

**Fig. 5:**
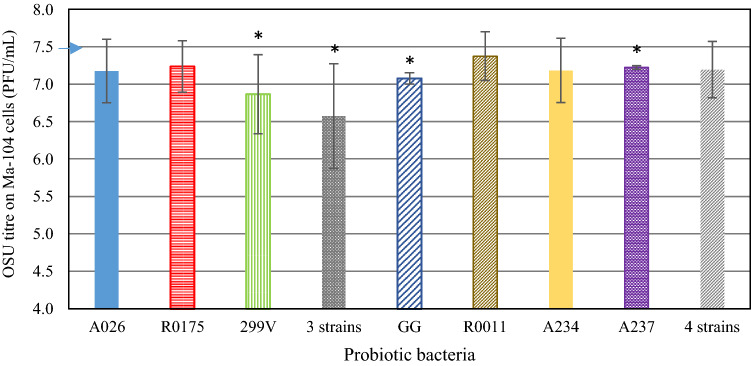
OSU infectivity (PFU/mL) in MA-104 cells after incubation with probiotic bacteria for 16 h (1 × 10^8^ CFU/mL) before removal of bacteria by centrifugation and filtration (3 strains: A026, R0175, 299V; 4 strains: GG, R011, A234, A237) (Pre-incubation C). The arrow represents the OSU control incubated without probiotic bacteria (7.46 PFU/mL). Values represent the mean (± SEM) of three separate different experiments. * *p* <0.05

Incubating *B. longum* R0175 with rotavirus before infecting IPEC-J2 cells reduced the infection rate by 14% (*p* = 0.014) (Fig. 4). For all of the other bacterial strains, the corresponding reductions were less than 10%. Different results were obtained when the rotavirus was incubated with the seven strains of probiotic bacteria for 16 h before viability was evaluated on MA104 cells (Fig. 5). Unlike the case for IPEC-J2 cells, *B. longum* R0175 did not significantly reduce the infection rate on MA-104 cells. Incubation with strains *Lpb. plantarum* 299V (*p* = 0.011), *Lcb. rhamnosus* GG (*p* = 0.029), and *Lb. gasseri* A237 (*p* = 0.020), as well as with the combination of strains *B. animalis lactis* A026, *B. longum* R0175, and *Lpb. plantarum* 299V (*p* = 0.014), produced a significant but moderate reduction in viral titres.

## Discussion

In recent years, there has been a growing interest in the use of probiotics to reduce symptoms of intestinal infections, which has led to the development of a variety of commercially available products for human and animal dietary supplementation. Probiotics or their metabolites can act on viral particles to impair infectivity [9, 37]. They can adhere to the pathogen and interfere with colonization by inactivating the pathogen or preventing its adhesion. The strain *B. longum* BORI was isolated from healthy infants and is considered safe to use to treat children with rotavirus infections. In many countries, it can be used in foods or as a probiotic supplement [20, 38]. Han et al. [37] incubated a whole bacterial cell extract of *B*. *longum* BORI with rotavirus WA before infecting MA104 cells and found a considerable reduction in infectivity. These authors indicated that low-molecular-weight and non-proteinaceous components derived from *B*. *longum* BORI appeared to be responsible for the anti-rotaviral activity. Fernandez-Duarte et al. [18] reported that the infection rate at which the rotavirus RRV strain (rhesus monkey) infected MA-104 cells was reduced when the cells were previously exposed to probiotic bacteria to prevent the virus from entering the cells. Out of 10 strains tested, the most effective ones were *Lcb. casei*, *Limosilactobacillus (Lil.) fermentum*, *B. adolescentis,* and *B. bifidum*, with corresponding reduction rates of 31%, 37%, 42%, and 24%, respectively. Their results also showed that protein extracts from *Lcb. casei* and *B. adolescentis* could prevent adhesion of virus particles to MA104 cells. In this study, after 16-h pre-treatment of rotavirus OSU with probiotics, *B*. *longum* R0175 was the most efficient at reducing virus infection of IPEC-J2 cells. *Bifidobacterium animalis lactis* A026, *Lpb*. *plantarum* 299V, and *Lcb*. *paracasei* A234 showed a smaller reduction. For MA104 cells, no reduction in infectivity was observed with *B*. *longum* R0175 after 16 h of incubation with rotavirus; however, *Lpb*. *plantarum* 299V, *Lcb*. *rhamnosus* GG, and *Lb*. *gasseri* A237 showed a significant reduction. Although the strains used in this study seem less effective than some others reported on in the literature, it should be kept in mind that most studies using probiotics do not evaluate the effect of these bacteria on acidification and cytotoxicity. It is unknown whether acidification of the medium, on its own, can reduce viral infectivity. With *B*. *longum* R0175, results obtained for virus pre-treatment with probiotics followed by infection of IPEC-J2 and MA104 cells suggest that the probiotic bacteria prevented infection by blocking the rotavirus but did not inactivate the virus. These results also support the argument put forward in other studies, namely that the presence of probiotics in the cell environment interferes directly with the virus before viral challenge, thus mitigating the level of infection [18, 39].

Some other mechanisms of probiotics of action can also influence the course of viral infections by stimulating an innate immune reaction in cells that triggers the synthesis of proteins, cytokines, and mucin production and thus helps to preserve the integrity of the intestinal mucosa [2, 9]. In an environmentally acquired infection of rotavirus and *Escherichia coli*, after suckling piglets that tested positive for rotavirus were administered 10^9^ CFU of *B. lactis* HN019, the animals suffered less-severe diarrhea and showed a decrease in fecal rotavirus levels [40]. Results from the measurement of blood cell phagocytosis, the lymphocyte proliferative response, and fecal anti-rotavirus antibodies suggested that an immune-mediated response occurred in probiotic-fed piglets, but not in control animals. Recent *in vitro* studies showed that *B*. *infantis* MCC12 and *B*. *breve* MCC1274 were effective in stimulating the innate immune response of bovine intestinal cells and porcine intestinal epithelial cells, thereby increasing their resistance to rotavirus OSU infection [16, 41]. Thompson et al. [39] analyzed changes in expression of 44 genes from bovine intestinal epithelial cells (BIEC) over time (2 h to 12 h) in the presence or absence of *Lcb. plantarum* 299V to gain a better understanding of the innate immune response. The results indicated that genes involved in the innate immune response were expressed. The response was relatively stable over time, and it seemed to prepare cells to react to infection. These authors also subjected the probiotic-pre-treated BIEC to a bovine rotavirus challenge. Analysis of the gene expression responsible for the innate immune response combined with plaque assay to determine virus viability showed that cells with probiotic pre-treatment were better prepared to counter infection, as this treatment stimulated a response limiting growth of the virus. For the strains evaluated in our *in vitro* assays, the results demonstrated that *B. longum* R0175, *B. animalis lactis* A026, and *Lpb. plantarum* 299V acted on IPEC-J2 cells, making them more resistant to rotavirus, either by stimulating the native immune response or by blocking virus attachment. Further studies of gene expression in IPEC-J2 cells in the presence of these probiotic strains and under certain pre-treatment conditions could provide a better understanding of the mechanisms behind the protective effect of certain probiotic bacteria and their role in reducing the rotavirus OSU infection rate.

The intestinal epithelium constitutes a barrier that protects the host against pathogenic microorganisms. Cell membranes have lipid rafts, which are sections of the membrane that are rich in cholesterol and sphingolipids. Cholesterol has been found to play a role in cell infection by rotavirus. This is evidenced by the reduction in infectivity that occurs after cholesterol is removed through the use of chelating agent methyl-ꞵ-cyclodextrin and by the increase in infectivity that occurs after cholesterol is replenished in the media [42–44]. For our study, optimization of rotavirus OSU replication conditions with IPEC-J2 cells had to be performed to obtain sufficiently high infection levels that would enable us to determine the impact of probiotic pre-treatments on viral infection in an accurate manner. The addition of an aqueous cholesterol cell culture solution to the maintenance medium during infection of IPEC-J2 cells appeared to play an important role in increasing the total level of viral infection.

Among the numerous applications of flow cytometry (FC), one study [45] reported that FC was used to detect rotavirus in MA-104 and Caco-2 cells in water samples. Bosch et al. [46] established a protocol for the detection of rotavirus Wa in CaCo-2 cells. Barardi et al. [47] used FC to detect simian rotavirus in MA-104 cells and artificially inoculated oysters. Yan et al. [48] used flow cytometry to confirm the growth of porcine circovirus in IPEC-J2 cells. In this study, flow cytometry allowed us to discriminate between cells and debris and thus obtain an accurate count of all of the cells in each sample. The method could also be used to distinguish infected cells from uninfected cells in a sample (specifically by counting infected cells). Since each probiotic treatment of OSU-infected cells was compared with a control involving the same probiotic treatment without OSU infection, the zone of uninfected cells in a sample could be determined for each treatment, bringing out the zone of infected cells. Using flow cytometry, it was possible to quantify rotavirus-OSU-infected IPEC-J2 cells rapidly, precisely and efficiently, which is not the case for other classical fluorescence techniques.

Since the beneficial effect of probiotics on the host appears to be strain-specific [9, 10], each strain needs to be assayed separately for selection purposes. The *in vitro* models can provide information on new strains. Results from this study reveal that *B. longum* R0175, *B*. *animalis lactis* A026, and *Lpb. plantarum* 299V can act to protect cells before infection. Furthermore, *B. longum* R0175 demonstrated a significant capacity to reduce the infection rate when incubated with the virus before viral infection of the cells. For human and animal applications, probiotics or probiotic extracts must demonstrate stability when exposed to the harsh conditions of the gastric environment. Lactic acid bacteria (LAB) such as *Lactobacillus* and *Bifidobacterium* are known to resist bile acids [49, 50]. Preparations of probiotics intended for use with animal feeds are already available on the market and point to the value of conducting feasibility studies for the development of commercial applications of other validated strains [51]. However, further *in vitro* and *in vivo* studies on these strains are required to determine the conditions needed to ensure their optimal effectiveness as probiotics that can be used in animal feed to counter intestinal infections caused by rotaviruses.

## Conclusion

The platform using porcine enterocytes (IPEC-J2) combined with flow cytometry quantification represents a powerful tool to evaluate and compare the effectiveness of probiotic bacteria in preventing or alleviating porcine rotavirus infection. Moreover, the cell-probiotics-virus platform combined with cytometry analysis could be used in the future to analyze the immune response of IPEC-J2 cells (cytokine receptor, surface protein expressions, biomarkers, and apoptosis). Results from this study provide insight into probiotic strains that can be used to benefit animal health and to prevent or better control intestinal infections that cause diarrhea in farm animals. The efficient probiotic strains identified in this study could be used in further research undertaken to elucidate the mechanisms behind the reduction in the rotavirus OSU infection rate observed in IPEC-J2 cells. The antiviral levels noted in this study may not be sufficient to prevent infection by rotaviruses. Nevertheless, probiotics could be combined with other antiviral strategies to protect against diseases, as has been demonstrated for bacterial infections [52]. The findings of this study suggest that some probiotic strains act by blocking rotavirus infection. Further studies are needed to identify the bioactive compounds involved and to enhance their levels in probiotic cells.

## Supplementary Information

Below is the link to the electronic supplementary material.

Supplementary file1 (DOCX 381 KB)

Supplementary file2 (DOCX 331 KB)

## Data Availability

The datasets generated during and/or analyzed during the current study are available from the corresponding author on reasonable request.
