# Supplementary material for: Effect of probiotic bacteria on porcine rotavirus OSU infection of porcine intestinal epithelial IPEC-J2 cells
Source: Arch Virol. 2022 Jul 6;167(10):1999–2010. doi: 10.1007/s00705-022-05510-x (PMC9402510; doi:10.1007/s00705-022-05510-x)

**Figure S1** Graphic representation of AlexaFluor-647 labeled IPEC-J2 cells analyzed using cytometry analysis after 4 h infection with rotavirus OSU: (A, B, C) non-infected and (D, E, F) infected cells. The region of interest P4 (A, D) applied on SSC-A versus FSC-A graph (B, E) then applied on a PI-A versus AF647-A graph (C, F) on which the region of interest to infected cells was created (H1-UL: non-infected cells, H1-UR: infected cells). Positive rotavirus infected cells were determined as the number of cells exceeding the AF-647 fluorescence of non-infected cells treated simultaneously under the same experimental conditions. Results were expressed as a percentage of AF-647 positive cells in a sample


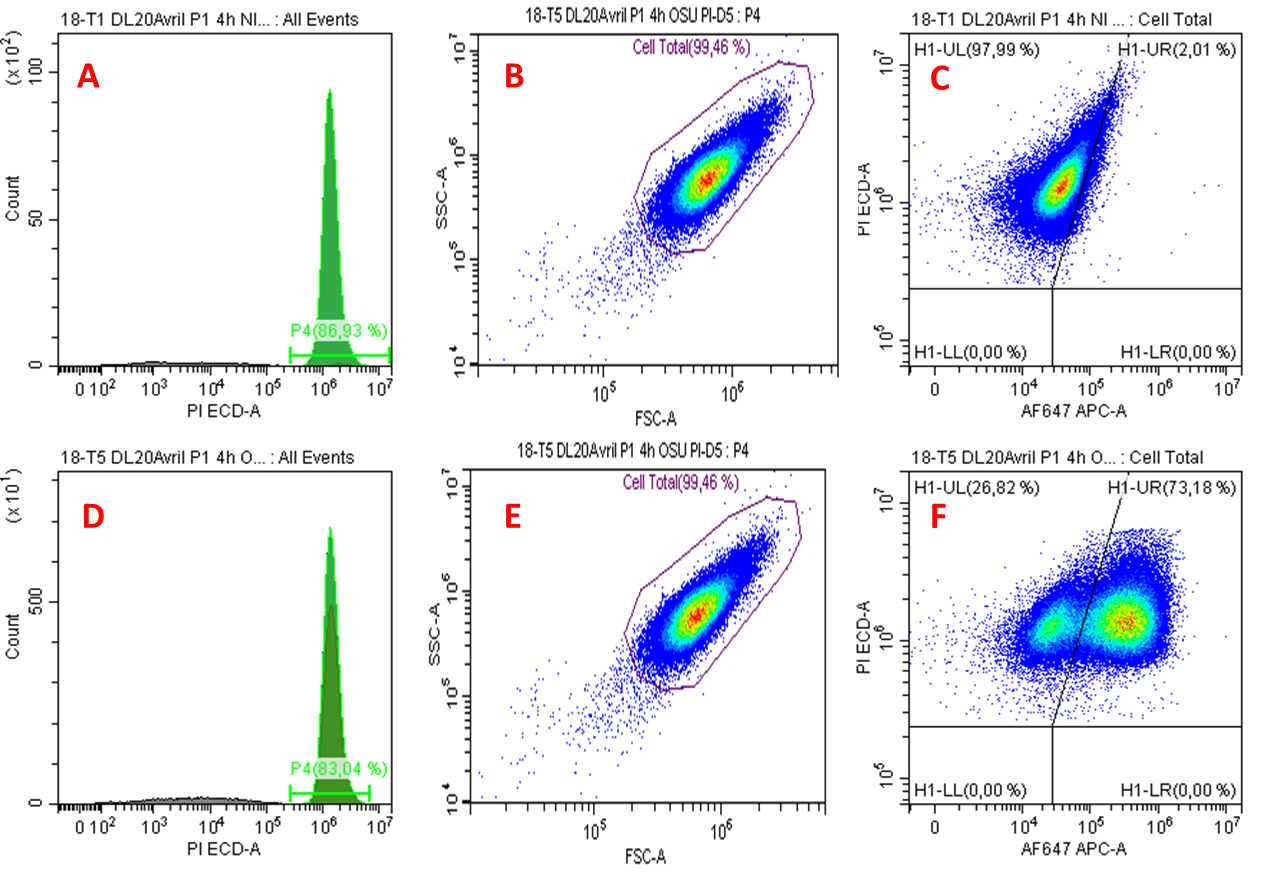

Supplement: Supplementary file 1 — Supplementary file1 (DOCX 381 KB) [file 705_2022_5510_MOESM1_ESM.docx]
