# Supplementary material for: Effect of probiotic bacteria on porcine rotavirus OSU infection of porcine intestinal epithelial IPEC-J2 cells
Source: Arch Virol. 2022 Jul 6;167(10):1999–2010. doi: 10.1007/s00705-022-05510-x (PMC9402510; doi:10.1007/s00705-022-05510-x)
Supplement: Supplementary file 2 — Supplementary file2 (DOCX 331 KB) [file 705_2022_5510_MOESM2_ESM.docx]

**Figure S2** Infection rate of IPEC-J2 infected cells with OSU (%) pre-treated 1 h with probiotic bacteria at 1 X 10^8^ and 5 X 10^8^ CFU/ml (Pre-treatment B). 3S: mix of the 3 strains. Values represent means (±SEM) of 3 different experiments
